# Increased functional connectivity between default mode network and visual network potentially correlates with duration of residual dizziness in patients with benign paroxysmal positional vertigo

**DOI:** 10.3389/fneur.2024.1363869

**Published:** 2024-03-04

**Authors:** Zhengwei Chen, Yaxian Cai, Lijie Xiao, Xiu-E Wei, Yueji Liu, Cunxin Lin, Dan Liu, Haiyan Liu, Liangqun Rong

**Affiliations:** ^1^Department of Neurology, Second Affiliated Hospital of Xuzhou Medical University, Xuzhou, Jiangsu, China; ^2^Department of Neurology, General Hospital of the Yangtze River Shipping, Wuhan, Hubei, China; ^3^Graduate School of Xuzhou Medical University, Xuzhou, Jiangsu, China

**Keywords:** benign paroxysmal positional vertigo, residual dizziness, resting-state fMRI, static functional network connectivity, dynamic functional network connectivity, independent component analysis

## Abstract

**Objective:**

To assess changes in static and dynamic functional network connectivity (sFNC and dFNC) and explore their correlations with clinical features in benign paroxysmal positional vertigo (BPPV) patients with residual dizziness (RD) after successful canalith repositioning maneuvers (CRM) using resting-state fMRI.

**Methods:**

We studied resting-state fMRI data from 39 BPPV patients with RD compared to 38 BPPV patients without RD after successful CRM. Independent component analysis and methods of sliding window and k-means clustering were adopted to investigate the changes in dFNC and sFNC between the two groups. Additionally, temporal features and meta-states were compared between the two groups. Furthermore, the associations between fMRI results and clinical characteristics were analyzed using Pearson’s partial correlation analysis.

**Results:**

Compared with BPPV patients without RD, patients with RD had longer duration of BPPV and higher scores of dizziness handicap inventory (DHI) before successful CRM. BPPV patients with RD displayed no obvious abnormal sFNC compared to patients without RD. In the dFNC analysis, patients with RD showed increased FNC between default mode network (DMN) and visual network (VN) in state 4, the FNC between DMN and VN was positively correlated with the duration of RD. Furthermore, we found increased mean dwell time (MDT) and fractional windows (FW) in state 1 but decreased MDT and FW in state 3 in BPPV patients with RD. The FW of state 1 was positively correlated with DHI score before CRM, the MDT and FW of state 3 were negatively correlated with the duration of BPPV before CRM in patients with RD. Additionally, compared with patients without RD, patients with RD showed decreased number of states and state span.

**Conclusion:**

The occurrence of RD might be associated with increased FNC between DMN and VN, and the increased FNC between DMN and VN might potentially correlate with the duration of RD symptoms. In addition, we found BPPV patients with RD showed altered global meta-states and temporal features. These findings are helpful for us to better understand the underlying neural mechanisms of RD and potentially contribute to intervention development for BPPV patients with RD.

## Introduction

Benign paroxysmal positional vertigo (BPPV), also known as canalithiasis, is characterized by transient vertigo and nystagmus induced by positional change, accompanied by nausea and vomiting in severe cases (1, 2). BPPV is the most common vertigo disease in clinical practice, accounting for 17–42% of all vertigo diseases (3). Canalith repositioning maneuvers (CRM) are the most effective treatment for BPPV (4, 5). However, after successful CRM, 31–60% of BPPV patients still report dizziness and discomfort (6), manifested as non-rotating dizziness, walking instability, disorientation, floating sensation, fogginess, or drowsiness without positional vertigo and nystagmus, these symptoms are called residual dizziness (RD) (7, 8).

So far, despite the neural mechanisms of RD are still not clear, recent studies have provided some promising insights with the application of resting-state functional magnetic resonance imaging (rs-fMRI). In a recent rs-fMRI study, methods of fractional amplitude of low-frequency fluctuations (fALFF) and regional homogeneity (ReHo) were used to compare the differences in brain function between patients with BPPV and healthy volunteers. The authors reported increased functional activities in bilateral pons and left posterior cerebellar regions in BPPV patients, these results indicated that the functional changes in pons might be related to RD after CRM (9). In another rs-fMRI study, Fu et al. applied fALFF analysis to observe the differences in brain functional activity between RD patients and non-RD patients after CRM, the authors found decreased functional activity in bilateral precuneus in patients with RD compared to patients without RD (10). The above two studies preliminarily suggested that the occurrence of RD symptoms in BPPV patients might be related to central integration and compensation. However, they only focused on local brain regions of cerebellum, brainstem and precuneus. In fact, the human brain is actually a complex system carried out by networks which are composed of multiple brain regions, and the functional connectivity among these networks changes dynamically with time-varying effects during fMRI scanning (11–13). Therefore, it is more valuable to further investigate brain functional network connectivity (FNC) patterns and dynamic characteristics in BPPV patients with RD after CRM.

In the present study, images of rs-fMRI, as well as demographic, clinical characteristics and behavioral scales of BPPV patients with and without RD symptoms were collected. Static FNC (sFNC) and dynamic FNC (dFNC) methods were adopted. We aimed to explore the differences in sFNC and dFNC between BPPV patients with and without RD symptoms after successful CRM, and to investigate the associations between neuroimaging results and clinical manifestations in patients with RD. The full recovery of vestibular symptoms requires adequate vestibular compensation (14–16). We speculate that RD is associated with inadequate vestibular compensation of the central nervous system, abnormal functional reorganization of relevant brain networks may occur. Furthermore, we expect the altered sFNC or dFNC may correlate with the duration of RD symptoms in patients with BPPV.

## Materials and methods

### Participants

BPPV was diagnosed based on the diagnostic criteria established by Bárány Society in 2015 (17). In this study, only patients with unilateral posterior semicircular canal BPPV were included (identified by Dix–Hallpike test) (18). All BPPV patients were right-handed, without other neurological, psychiatric, or systemic diseases. Patients with alcohol or drug abuse were excluded. Patients with secondary BPPV caused by vestibular migraine (VM), vestibular neuritis (VN), Meniere’s disease (MD) or other vestibular disorders were excluded. Patients with MRI contraindications or the presence of irremovable metallic substances affecting the quality of fMRI images were excluded. All patients underwent routine MRI (T1WI + T2WI + DWI + FLAIR) scans to rule out occult central nervous system diseases. In addition, patients with moderate–severe white matter hyperintensity on FLAIR images were excluded.

All BPPV patients were treated with CRM (Epley or Semont) after a definite diagnosis (19, 20). We repeated the Dix–Hallpike test 60 min after CRM to ensure a successful CRM (patients reported no positional vertigo and no positional nystagmus were observed). Demographic and clinical characteristics were collected, including age, gender, years of education, affected side, duration of vertigo before successful CRM, and scores of vertigo visual analog scale (VVAS) and dizziness handicap inventory (DHI) before successful CRM. All patients were followed up on the seventh day after successful CRM by a face to face interview. Patients again received diagnostic positional tests. All patients received tests of Montreal Cognitive Assessment (MoCA), Hamilton Anxiety Scale (HAMA) and Hamilton Depression Scale (HAMD) during the follow-up. Patients with scores of MoCA<26, HAMA>14 or HAMD>17 were excluded. For patients with negative diagnostic positional tests, if a patient reported RD symptoms lasting more than 5 days on the day of the follow-up visit, he or she would be assigned to the RD group. On the other hand, if a patient reported no RD symptoms from successful CRM to the day of the follow-up visit, he or she would be assigned to the non-RD group. All patients in the RD and non-RD group would be scanned by rs-fMRI within the next 2 days. In addition, patients in the RD group received tests of dizziness visual analog scale (DVAS) and DHI after successful CRM. Furthermore, the duration of RD symptoms was recorded by follow-up (the follow-up period was 90 days).

Totally, 43 BPPV patients with RD and 45 patients without RD were recruited from the Department of Neurology of the Second Affiliated Hospital of Xuzhou Medical University between September 2020 and February 2023.

### MRI data acquisition

All patients were scanned using a 3.0 T MRI system (GE DISCOVERY 3.0 T magnetic resonance instrument with the 8-channel cranial coil, United States). Patients were required to keep their eyes closed, keep their heads still, and to stay relaxed and not to fall asleep. Images of rs-fMRI were collected using an echo-planar imaging sequence and each functional data contained 210 volumes (time points). The parameters were as follows: repetition time (TR) = 2000 ms, echo time (TE) = 30 ms, flip angle (FA) = 90°, field of view (FOV) = 200 × 200 mm, matrix = 64 × 64, thickness = 3.6 mm, gap =0 mm. We adopted a 3D-BRAVO sequence to collect 3-dimensional high-resolution T1-weighted images (TR = 2,500 ms, TE = 3.5 ms, FA = 8°, matrix = 256 × 256, thickness = 1 mm, number of slices = 156).

### MRI data preprocessing

Based on MATLAB 2016a (Mathworks, Natick, MA, United States), GRETNA (v2.0.0)^1^ and Statistical Parametric Mapping 12 (SPM12)^2^ were used to preprocess rs-fMRI data. The DICOM format images were first converted to NIFTI format. Then the first 10 time points were removed to reduce initial unstable blood oxygen level-dependent (BOLD) signal. For the remaining 200 time points, slice timing and head motion correction were performed to guarantee that all voxels within one time point had been acquired at the same time and to lessen the influence of head motion on fMRI images. In the present study, functional data with head motion exceeded 2 mm [displacement distance (x, y, z)] or 2° [rotation angle (x, y, z)] were removed. Then, T1-weighted images were converted to NIFTI format, and subsequently were segmented into white matter, gray matter and cerebral spinal fluid. The segmented T1 images were normalized to Montreal Neurological Institute (MNI) and resampled at a resolution of 3 mm × 3 mm × 3 mm. The normalized data were smoothed with a Gaussian kernel of 6 mm full-width at half maximum. Four patients in RD group and seven patients in non-RD group were excluded due to the criterion of head motion correction. Finally, the remaining 39 patients in RD group and 38 patients in non-RD group were included in the following analysis.

### Group independent component analysis

To create resting state networks (RSNs), GICA was performed using GIFT toolbox (GIFTv3.0b).^3^ The preprocessed functional data were dimension-reduced using principal components analysis (PCA) at the subject level to decrease computational complexity, followed by decomposition of the concatenated subject-reduced functional data in the group level including all patients. Minimum description length (MDL) criterion was adopted to compute the number of independent components (ICs) to be 25 (21). To guarantee the stability and reproducibility of ICs, we repeated the Infomax algorithm 100 times in ICASSO (22, 23).^4^ Eventually, GICA was carried out to back-reconstruct the subject-specific spatial maps and time courses of each IC (24).

### Identification of resting-state networks

ICs were identified as significant RSNs based on previous studies (25, 26) and the follow criterions: (1) we manually observed whether the peak activations of an IC were located primarily in grey matter; If that is the case, the peak activations should not show spatial overlap with vascular, ventricular or susceptibility artifacts. (2) The time courses of an IC should show low-frequency fluctuations (the ratio of power below 0.10 Hz, or the ratio of power between 0.15 Hz and 0.25 Hz). (3) Power_LF_/Power_HF_ (low frequency to high frequency power ratio) ≥ 3. Among 25 ICs, we identified 13 meaningful ICs. These 13 ICs were then classified into 8 RSNs based on the spatial correlation values between ICs and the RSNs templates and by visual observation.

### Static functional network connectivity

After GICA and the identification of RSNs, the Mancovan toolbox (v1.0) in GIFT software was adopted to compute the correlations between any two RSNs for each patient. Before calculating sFNC, we applied additional post-processing steps on time courses to remove the noise and artifacts, mainly including (1) detrending of linear, quadratic, and cubic trends; (2) despite time courses; (3) analyzing the 6 realignment parameters and their derivatives by multiple regression (27); and (4) low-pass filtering with a cutoff frequency of 0.15 Hz was applied, which was performed on the TC of ICs. Afterwards, we calculated the Pearson correlation coefficient (r) between each summary time course and other summary time courses and converted r value into z value by Fisher-Z transformation. Finally, a 13 × 13 FNC matrix for each patient was generated.

### Dynamic functional network connectivity

The temporal dFNC, a module of GIFT software (dFNC v1.0a), was applied to compute Pearson’s correlations between time courses of ICs in dynamic states. Before calculating dFNC, we applied the same post-processing steps as described in sFNC section. Then, we used a sliding time window approach with a window size set to 30 TRs and a Gaussian (σ = 3 TRs) to divide the time courses of all ICs, and sliding 1TR at each step (28, 29). After that, the k-means clustering method (distance method of square Euclidean, 500 iterations and 150 repeats) was employed to assign the windowed FNCs of all patients into a set of states (30, 31). Based on an elbow criterion (31), the number of optimal clusters was set to 5. Three temporal properties (fractional windows, mean dwell time and number of transitions) and four meta-states (number of states, change between states, state span and total distance) of dFNC for BPPV patients with and without RD were also calculated. Fractional windows refer to the time spent in each state as a percentage of the total time. Mean dwell time means the average time the participants spent in a certain state. Number of transitions represents the number of times a subject switched between different states. Number of states refers to the number of distinct meta-states subjects occupy during the scan length. Change between states means the number of times that participants switch from one meta-state to another. State span represents the range of meta-states subjects occupy. Total distance refers to the overall distance traveled by each subject through the state space.

## Statistical analysis

### Analysis of demography and clinical characteristics

The IBM SPSS 22.0 software package was applied to compare the differences in demography and clinical characteristics of BPPV patients with and without RD. The two-sample t-tests were used for parametric continuous variables (age, years of education, scores of MoCA, HAMA, HAMD, VVAS and DHI, and duration of vertigo before successful CRM) and chi-square tests were used for categorical variables (gender and affected side). A *p* < 0.05 was considered significant.

### Differences between groups in sFNC

The two-sample t-tests implanted in the Mancovan toolbox was performed to analyze group differences in sFNC with a significance threshold of *p* < 0.05 [false discovery rate (FDR) corrected], controlling for age, years of education, scores of MoCA, HAMA, HAMD, VVAS and DHI, and duration of vertigo before successful CRM.

### Differences between groups in dFNC

To investigate group differences in FNC in each state, the two-sample t-tests included in temporal dFNC sub-package was carried out. Age, years of education, scores of MoCA, HAMA, HAMD, VVAS and DHI, and duration of vertigo before successful CRM were included as covariates. The results were corrected for multiple comparisons using FDR (*p* < 0.05). In addition, to compare the three temporal properties and four meta-states of dFNC between BPPV patients with and without RD, the two-sample t-tests were employed. Age, years of education, scores of MoCA, HAMA, HAMD, VVAS and DHI, and duration of vertigo before successful CRM were included as covariates. A *p* < 0.05 (FDR corrected) was considered significantly different.

### Brain-behavioral correlation analysis

For sFNC and dFNC showing significant between-group differences (two-sample t-test, *p* < 0.05, FDR corrected), we performed Pearson’s partial correlation analysis between altered rs-fMRI (including *z*-values of the altered sFNC, *z*-values of the altered dFNC in each state, three temporal properties and four meta-states) and clinical characteristics (duration of vertigo, scores of VVAS and DHI before successful CRM, and duration of RD, scores of DVAS and DHI after successful CRM) in BPPV patients with RD, controlling for age, gender, educational years, MoCA, HAMA, HAMD and the affected side (*p* < 0.05, FDR correction).

## Results

### Demographic and clinical characteristics

The demographic and clinical characteristics of BPPV patients with and without RD were summarized in Table 1. There was no obvious difference between the two groups in age, gender, educational years, affected side, scores of MoCA, HAMA, HAMD and VVAS before successful CRM (all *p* > 0.05). Despite this, we found longer duration of vertigo (*p* = 0.006) and higher scores of DHI (*p* = 0.008) before successful CRM in patients with RD by contrast with patients without RD.

**Table 1:** Demographic and clinical characteristics of patients with and without RD.

	Without RD (*n* = 38; Mean ± SD)	RD (*n* = 39; Mean ± SD)	Value of *p*
Age (years)	51.71 ± 8.44	55.00 ± 8.53	0.093
Gender (female/male)	27/11	34/5	0.081
Education (years)	11.18 ± 3.20	10.00 ± 3.42	0.121
MoCA	28.05 ± 1.37	27.79 ± 1.22	0.386
HAMA	7.39 ± 2.11	8.36 ± 2.43	0.068
HAMD	8.08 ± 2.27	9.10 ± 3.07	0.101
Affected side (left/right)	22/16	18/21	0.303
Before successful CRM			
Duration of vertigo (days)	2.86 ± 2.36	4.63 ± 3.11	0.006
VVAS	5.76 ± 2.06	6.59 ± 2.19	0.092
DHI	38.08 ± 11.60	46.64 ± 15.74	0.008
After successful CRM			
Duration of dizziness (days)	/	22.41 ± 15.14	/
DVAS	/	2.95 ± 1.02	/
DHI	/	25.08 ± 10.33	/

RD, Residual dizziness; MoCA, Montreal cognitive assessment scale; HAMA, Hamilton anxiety scale; HAMD, Hamilton depression scale; CRM, *Canalith repositioning maneuvers; VVAS, Vertigo* visual analog scale; DHI, Dizziness handicap inventory; DVAS, Dizziness visual analog scale.

### Results of resting-state networks

**Figure 1:**
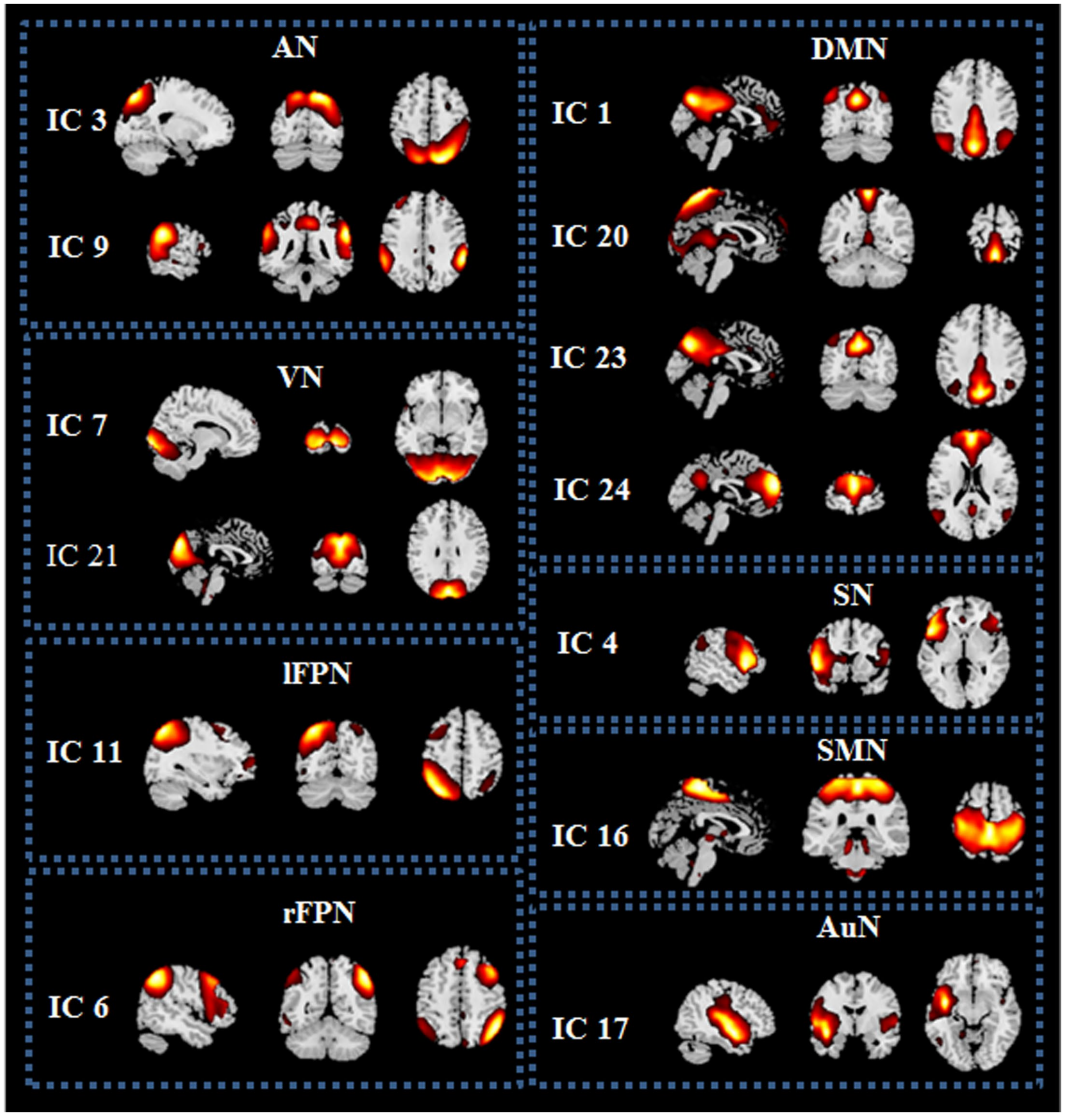
Spatial maps of the 13 independent components (ICs) identified as resting state networks (RSNs) from the 25 ICs. DMN, default mode network; lFPN, left frontoparietal network; rFPN, right frontoparietal network; SN, salience network; AN, attention network; VN, visual network; SMN, sensorimotor network; AuN, auditory network.

Spatial maps of all 13 ICs were shown in Figure 1. These 13 ICs were grouped into 8 RSNs, namely, default mode network (DMN; IC 1, IC 20, IC 23 and IC 24), left frontoparietal network (lFPN; IC 11), right frontoparietal network (rFPN; IC 6), salience network (SN; IC 4), attention network (AN; IC 3 and IC 9), visual network (VN; IC 7 and IC 21), sensorimotor network (SMN; IC 16) and auditory network (AuN; IC 17). The detailed information of ICs was displayed in Table 2.

**Table 2:** Peak activation information of the 13 independent components (ICs).

IC regions	Peak MNI coordinate			BA
	X	Y	Z	
Default mode network				
IC 1 L precuneus	−6.5	−57.5	8.5	23
IC 20 R precuneus	2.5	−54.5	71.5	7
IC 23 L posterior cingulate cortex	0.5	−74.5	36.5	7
IC 24 L medial prefrontal cortex	−0.5	59.5	18.5	10
Left frontoparietal network				
IC 11 L posterior parietal cortex	−33.5	−69.5	53.5	7
Right frontoparietal network				
IC 6 R posterior parietal cortex	50.5	−56.5	48.5	39
Salience network				
IC 4 L temporal pole	−53.5	15.5	−2.5	45
Attention network				
IC 3 R superior parietal lobule	20.5	−77.5	51.5	7
IC 9 R inferior parietal lobule	62.5	−41.5	33.5	40
Visual network				
IC 7 R Lingual gyrus	12.5	−98.5	−8.5	17
IC 21 L cuneus	0.5	−86.5	29.5	19
Sensorimotor network				
IC 16 R paracentral lobule	2.5	−33.5	63.5	4
Auditory network				
IC 17 L superior temporal lobe	42.5	−3.5	−12.5	22

IC, Independent component; BA, Brodmann area; L, left; R, right; MNI, Montreal Neurological Institute.

### Static functional network connectivity results

**Figure 2:**
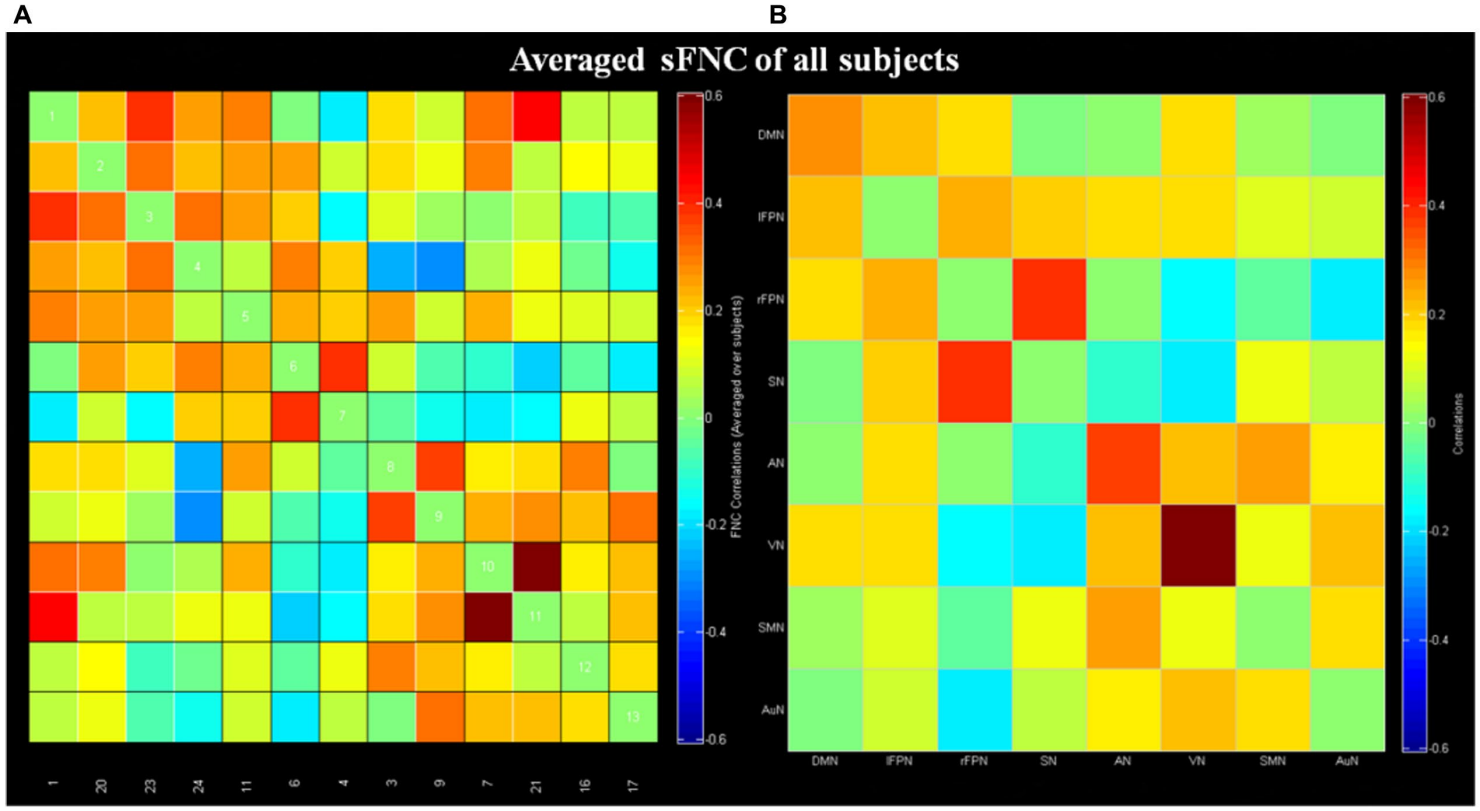
Results of static functional network connectivity (sFNC; one sample *t*-test, *p <* 0.05, FDR corrected). **(A)** The mean sFNC matrices of all subjects in the 13 independent components. **(B)** The averaged sFNC matrices of all subjects in eight networks. DMN, default mode network; lFPN, left frontoparietal network; rFPN, right frontoparietal network; SN, salience network; AN, attention network; VN, visual network; SMN, sensorimotor network; AuN, auditory network.

The averaged sFNC matrices of all subjects were presented in Figure 2 (one sample t-test, *p <* 0.05, FDR corrected). Compared to BPPV patients without RD, BPPV patients with RD displayed no obvious abnormal sFNC (two sample t-test, *p* > 0.05, FDR correction).

### Dynamic functional network connectivity results

**Figure 3:**
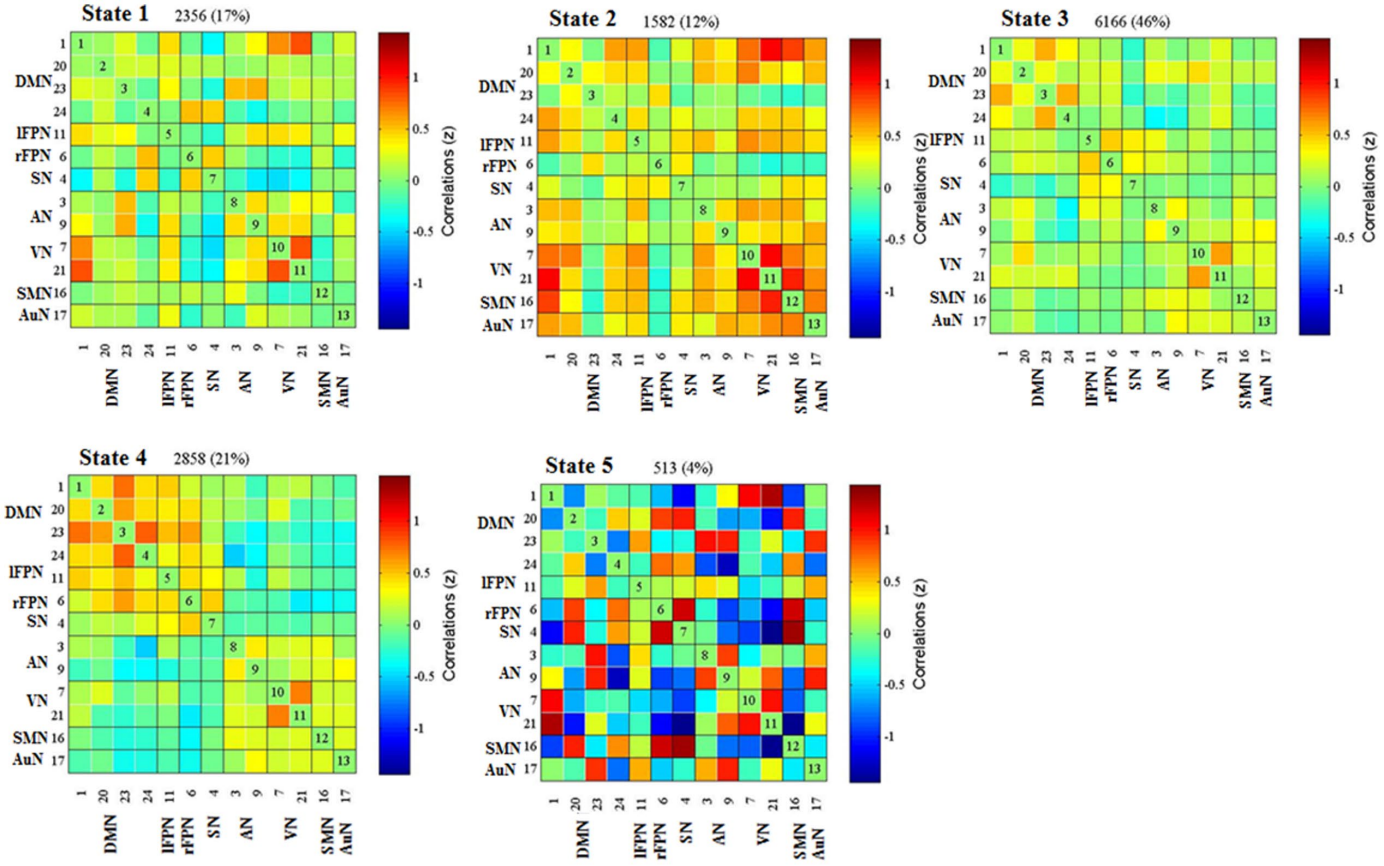
Results of the clustering analysis for each state. Percentage corresponds to the time that all subjects dwell in a certain state, and the order of the state corresponds to the order of the introduction of *k*-means algorithm. DMN, default mode network; lFPN, left frontoparietal network; rFPN, right frontoparietal network; SN, salience network; AN, attention network; VN, visual network; SMN, sensorimotor network; AuN, auditory network.

Cluster centroid of the five states and their respective occurrence frequency and percentage were displayed in Figure 3. It is worth noting that not all subjects have all five states. State 1 accounted for 17% of all windows and it contained 19 RD and 10 non-RD patients. State 1 was generally characterized by the existence of relatively strong positive between-network connectivity in DMN (IC 1)-VN (IC21) and within-network connectivity in VN (IC 7-IC 21). State 2, which accounted for 12% of all windows (13 RD and 13 non-RD patients), was featured by relatively strong positive between-network connectivity in SMN (IC 16)-VN (IC21) and within-network connectivity in VN (IC 7-IC 21). State 3 accounted for 46% (the largest occurrence frequency) and it contained 28 RD and 34 non-RD patients. State 3 was characterized by sparse and weak between-network and within-network connections. State 4 accounted for 21% and it was made up of 26 RD and 22 non-RD patients. State 4 was featured by relatively strong positive within-network connections in VN (IC 7-IC 21), and in DMN (IC 1-IC 23; IC 23-IC 24). State 5 accounted for 4% (the smallest occurrence frequency) and it was composed of 4 non-RD patients only. State 5 was distinguished by significant strong positive and negative between-network and within-network connections broadly.

**Figure 4:**
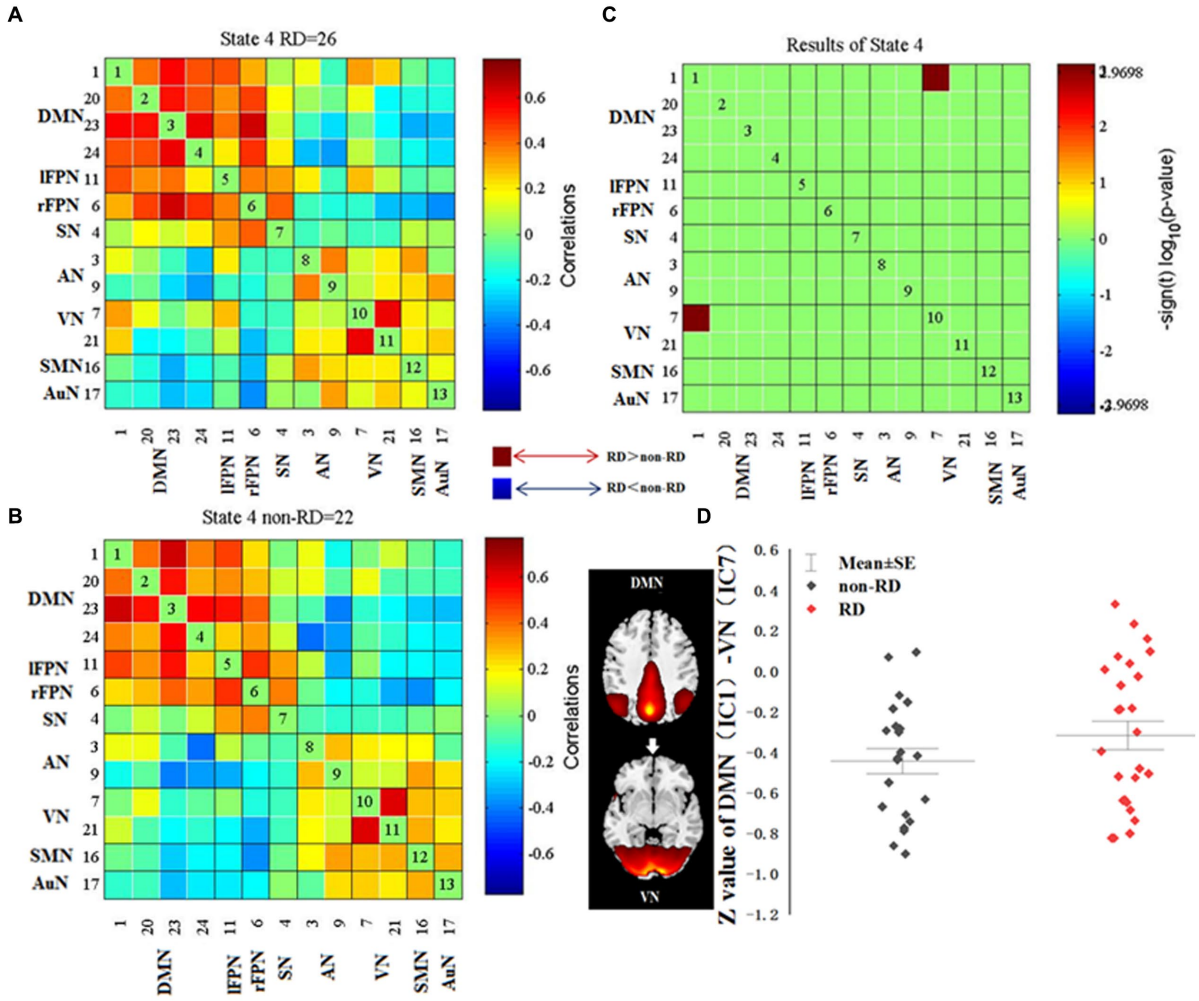
Differences in dynamic functional network connectivity (dFNC) between patients with and without residual dizziness (RD; *p* < 0.05, FDR corrected). **(A,B)** represent median FNC matrices, in RD and non-RD, respectively. The number of participants displayed in median FNC matrices were subjects who showed correlation for at least 10 windows and were included in the data to determine group differences. **(C)** represents difference between **A** and **B**. Red means RD > non-RD, blue indicates RD < non-RD. **(D)** shows difference in *z* value of DMN (IC 1)-VN (IC 7) between RD and non-RD patients in state 4. DMN, default mode network; lFPN, left frontoparietal network; rFPN, right frontoparietal network; SN, salience network; AN, attention network; VN, visual network; SMN, sensorimotor network; AuN, auditory network.

For each state, the FNC between patients with and without RD were compared. Significant differences were found in FNC between patients with and without RD in state 4. As displayed in Figure 4, compared with patients without RD, patients with RD showed increased FNC between DMN (IC 1) and VN (IC 7) in state 4 (*p* < 0.05, FDR corrected).

**Figure 5:**
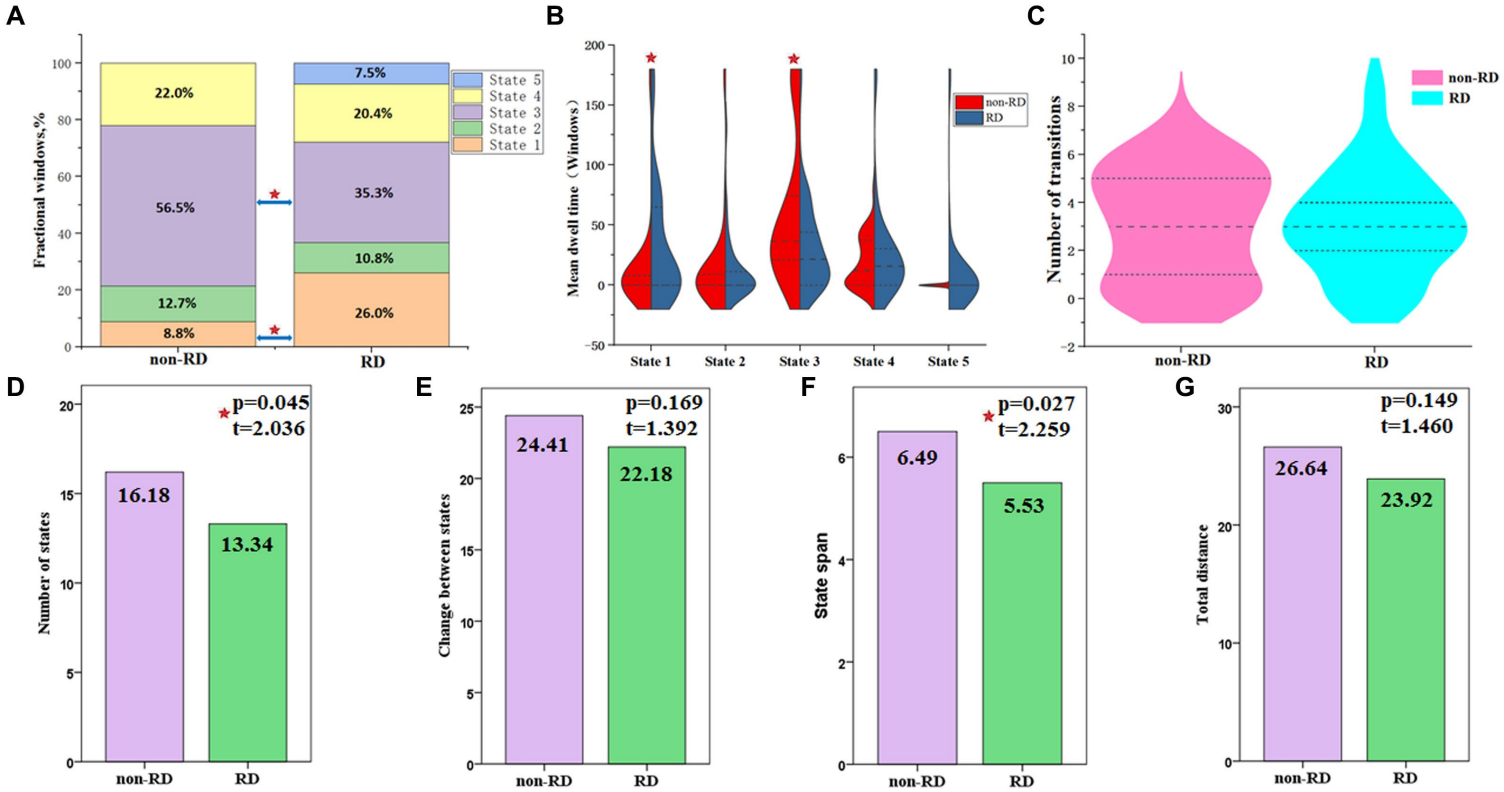
Temporal properties and meta-states of dynamic functional network connectivity for patients with and without residual dizziness (RD). **(A)** Percentage of total time patients spent in each state; **(B)** Mean dwell time; **(C)** Number of transitions between states; **(D)** Number of states; **(E)** Change between states; **(F)** State span; **(G)** Total distance. Red five-pointed star represents significant difference between two groups (*p* < 0.05, FDR corrected).

The differences of three temporal properties and four meta-states of dynamic FNC between the two groups were exhibited in Table 3 and Figure 5. Compared with patients without RD, we found significantly increased mean dwell time and fractional windows in state 1 but decreased mean dwell time and fractional windows in state 3 in BPPV patients with RD (*p* < 0.05, FDR corrected). No significant group difference was found in transition number. In addition, compared with patients without RD, patients with RD showed decreased number of states and state span (*p* < 0.05, FDR corrected). No significant group effect was found in change between states and total distance.

**Table 3:** Differences in the three temporal properties between patients with and without residual dizziness (RD).

Properties	State	Without RD (*n* = 38) Mean ± SD	RD (*n* = 39) Mean ± SD	Value of *p*
Mean dwell time (windows)	1	12.87 ± 39.19	33.38 ± 49.28	0.047
	2	15.70 ± 41.18	12.32 ± 25.73	0.667
	3	61.27 ± 60.43	30.20 ± 35.51	0.007
	4	18.77 ± 20.92	25.50 ± 40.11	0.360
	5	0 ± 0	10.56 ± 39.60	0.105
Fractional windows	1	8.8 ± 23.1%	26.0 ± 35.9%	0.015
	2	12.7 ± 290%	10.8 ± 20.4%	0.743
	3	56.5 ± 33.9%	35.3 ± 34.3%	0.008
	4	22.0 ± 26.1%	20.4 ± 26.7%	0.796
	5	0 ± 0%	7.5 ± 25.8%	0.077
Number of transitions		3.1 ± 2.3	3.1 ± 2.2	0.962

### Relationship between dFNC and clinical characteristics

**Figure 6:**
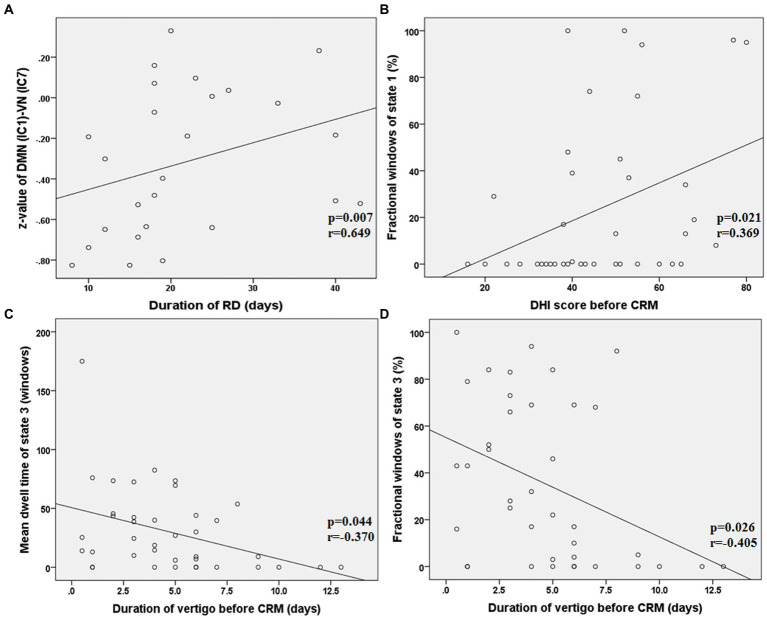
Results of the correlations between dFNC and clinical characteristics in patients with RD (all *p* < 0.05, FDR corrected). RD, residual dizziness; DMN, default mode network; VN, visual network; IC, independent component; DHI, dizziness handicap inventory; CRM, canalith repositioning maneuvers. **(A)** The *z*-value between DMN (IC 1) and VN (IC 7) was positively correlated with the duration of RD (*p* = 0.007, *r* = 0.649, FDR corrected); **(B)** The fractional windows of state 1 was positively correlated with the DHI score before CRM (*p* = 0.021, *r* = 0.369, FDR corrected); **(C)** The mean dwell time of state 3 was negatively correlated with the duration of vertigo before CRM (*p* = 0.044, *r* = −0.370, FDR corrected); **(D)** The fractional windows of state 3 was negatively correlated with the duration of vertigo before CRM (*p* = 0.026, *r* = −0.405, FDR corrected).

As shown in Figure 6, in state 4, the FNC (z-value) between DMN (IC 1) and VN (IC 7) was positively correlated with the duration of RD in patients with RD (*p* = 0.007, *r* = 0.649, FDR corrected). The fractional windows of state 1 was positively correlated with the DHI score before CRM in patients with RD (*p* = 0.021, *r* = 0.369, FDR corrected). The mean dwell time of state 3 was negatively correlated with the duration of vertigo before CRM in patients with RD (*p* = 0.044, *r* = −0.370, FDR corrected). The fractional windows of state 3 was negatively correlated with the duration of vertigo before CRM in patients with RD (*p* = 0.026, *r* = −0.405, FDR corrected).

## Discussion

To the best of our knowledge, the current study is the first one which adopted methods of data-driven ICA, sliding-time windows and k-means clustering to explore the changes in sFNC and dFNC in BPPV patients with RD after successful CRM. Compared with patients without RD, our results demonstrated that patients with RD showed altered dFNC. Specifically, RD patients showed increased FNC between DMN and VN in state 4, longer fractional windows and mean dwell time in state 1 and shorter fractional windows and mean dwell time in state 3, as well as decreased number of states and state span. In addition, we found that the altered dFNC were associated with certain clinical characteristics of patients with RD. These results indicated that alterations in dFNC were related to the presence of RD in BPPV patients after CRM. Our findings provided new insights into understanding the neural mechanism in BPPV patients with RD.

Previous studies have reported that a longer duration of BPPV and higher DHI score before successful treatment were related to the occurrence of RD and were considered as risk factors for RD (7, 32). In our study, patients with RD showed longer duration of BPPV and higher DHI score before successful CRM compare to patients without RD, these results were consistent with previous studies. Our results also demonstrated that patients with RD still suffered from RD symptoms lasting an average of 22 days. Therefore, it is import to explore the neural mechanism of RD for patients with BPPV.

### Altered dFNC between DMN and VN in patients with RD

In this study, no obvious alteration of sFNC was found between the two groups. However, in dFNC analysis, compared with non-RD patients, RD patients showed increased FNC between DMN (IC 1) and VN (IC 7) in state 4. These results indicated that dFNC was an extension of sFNC and could provide additional valuable information that might be missed in sFNC analysis (25).

The DMN is a “task-negative” network which can be divided into anterior DMN (aDMN) and posterior DMN (33, 34). The aDMN was reported to be mainly composed of medial prefrontal cortex (mPFC) and anterior cingulate cortex (ACC). The pDMN was constituted by precuneu (PCU) and posterior cingulate cortex (PCC) (33). Thus, the IC 24 (mPFC) that we identified in this study belonged to aDMN and the IC 1 (PCU), IC 20 (PCU) and IC 23 (PCC) were part of pDMN. It was considered that the aDMN was engaged in planning, integration and control while the pDMN was responsible for attention monitoring and self-centered cognition (33, 34). Functional changes in DMN have been reported in vestibular disorders of VM (35), VN (36) and persistent postural perceptual dizziness (PPPD) (37), indicating that DMN might be involved in vestibular information processing. In BPPV patients with RD, altered functional activities in PCU were observed by a previous resting-state fMRI study, which was consistent with the functional change of IC 1 (PCU) found in this study (10). The PCU, a key region of pDMN, was reported to be significantly activated during optokinetic stimulation (38) and visuo-spatial imagery (39). It was also reported that electrical stimulation of the PCU might evoke symptom of vertigo (40, 41). Thus, the pDMN might be involved in the integration of visual information and might play an important role in spatial positioning and perception.

The VN was reported to be grouped into primary VN (pVN) and secondary VN (sVN) (42, 43). The pVN is located in the medial cortex close to middle line, mainly including cuneus and lingual gyrus (42, 43). The sVN is mainly distributed in the extrastriate occipital cortex (42, 43). Thus, the IC 7 (lingual gyrus) and IC 21 (cuneus) that we identified in current study belonged to pVN. Previous rs-fMRI studies have reported the functional alterations of VN in patients with vertigo or dizziness symptoms, including PPPD (44), chronic unilateral vestibulopathy (CUVP) (45) and VM (35).

Our study revealed increased FNC between DMN and VN in BPPV patients with RD who experienced dizziness and imbalance after CRM. It was believed that visual and vestibular sensations, as well as proprioception are the three main elements of the human body to maintain balance (46, 47). In addition, it was reported that the impaired balance function during the onset of BPPV was mainly related to abnormal vestibular input (48). The complete recovery of vestibular symptoms requires adequate vestibular compensation in the central nervous system (14–16, 49). Sensory substitution is one of the strategies of central vestibular compensation. Visual sensation and proprioception are often the main sources of sensory substitution (15, 50, 51). Thus, we speculated that the increased FNC between DMN and VN might be associated with visual substitution, reflecting an enhanced perception, integration and processing of visual and spatial location information.

We also found that the FNC (z-score) between DMN and VN was positively correlated with the duration of RD in patients with RD. This result suggested that the increased FNC between DMN and VN was related to the delayed recovery of RD symptoms, indicating more time needed for visual substitution. In addition, since all rs-fMRI scans were performed on the 8th to 9th day of the onset of RD symptoms in patients with RD, and the duration of RD symptoms was obtained by follow-up after rs-fMRI scans, we suggested that the increased FNC (z-score) might potentially predict the duration of RD symptoms, but this suggestion should be further verified by a prediction analysis.

A recent MRI study discovered structural and functional changes in the cerebellum and pons in patients with BPPV, the authors indicated that the changes in pons function might be closely related to RD after CRM (9). In the present study, during the identification of RSNs, the IC 5 seemed to be the cerebellar network and the IC 12 seemed to belong to the brainstem network. But these two ICs failed to meet the criteria established in this study to identify a significant RSN. We recognized IC 5 and IC 12 as artifacts, not meaningful RNSs. Thus, the cerebellar and brainstem networks were not included in this study to analyze the functional changes in BPPV patients with RD. For all this, when focused on cortical networks, our study provided the direct evidence that the functional changes of DMN and VN were closely related to RD.

### Altered temporal properties of dFNC in patients with RD

The three temporal properties of dFNC were also analyzed. Although there was no significant diffidence between the two groups in transition numbers, compared with non-RD group, the RD group showed increased fractional windows and mean dwell time in state 1. The state 1 was characterized by relatively strong positive inter-network connectivity between DMN and VN, and relatively strong positive intra-network connectivity within VN. This result indicated that patients with RD were inclined to spend more time in a state in which more visual information was processed and integrated. In addition, we found that the fractional windows of state 1 were positively correlated with DHI score before CRM in patients with RD. That is to say, the more severe the vestibular symptoms of BPPV patients before successful CRM, the more likely they are to develop RD symptoms, and the longer they may stay in state 1 in which more visual information was processed and integrated. These results further confirmed that DMN and VN were closely related to the occurrence of RD.

State 3 was a sparse and weak connectivity state. Compared with modular connectivity state, regional connectivity state and strong connectivity state, it was reported that a dFNC state with sparse and weak connectivity was characterized by inefficient functional integration and less flexible interaction (52). Another result worth watching in this study was that patients with RD showed decreased fractional windows and mean dwell time in state 3. In addition, we found that the mean dwell time and fractional windows of state 3 were negatively correlated with the duration of vertigo before CRM in patients with RD. These results suggested that patients with RD were more likely to dwell in a state with more efficient functional integration and flexible interaction, and might reflect the functional plasticity and compensatory of these cortical networks after longer time repeated attacks of episodic vertigo.

### Altered meta-states of dFNC in patients with RD

We also calculated the four meta-states of dFNC, these meta-states were first reported by Miller. et al. (53). Compared with non-RD group, patients with RD exhibited decreased dynamic fluidity and dynamic range. In the aspect of dynamic fluidity, patients with RD entered fewer meta-states than patients without RD, as measured by number of states. In terms of dynamic range, patients with RD traveled through less state space than patients without RD, as measured by state span. The global meta-states have been proved to provide unique information in schizophrenia and fetal alcohol spectrum disorders (53–56). In this study, we first found that the global meta-states of dFNC could provide unique information for BPPV patients with RD. Unfortunately, we failed to find any significant relationship between the disrupted global meta-states and clinical features of RD patients. For all this, we believed that the decreased dynamic fluidity and dynamic range might be closely related to the occurrence of RD in patients with BPPV after successful CRM.

## Limitations

This study has certain limitations and shortcomings in some aspects. First, although the sample size met the requirements of statistics, we only included 39 patients with RD and 38 patients without RD. Subsequent studies with larger sample size and healthy control group are urgently needed. Second, brainstem and cerebellum networks were not included to analyze the functional changes in BPPV patients with RD, they might also play an important role in the development of RD. Finally, our study only adopted ICA method, future studies should combine ICA with other methods, for example, seed-based functional connectivity, graph theory analysis, etc.

## Conclusion

In summary, the present study revealed increased FNC between DMN and VN in BPPV patients with RD, the increased FNC (z-score) between DMN and VN might potentially correlate with the duration of RD symptoms. In addition, the current study discovered that BPPV patients with RD showed decreased dynamic fluidity and dynamic range. Furthermore, BPPV patients with RD spent more time in a state typified by strong positive inter-network connectivity between DMN and VN, and strong positive intra-network connectivity within VN. Additionally, BPPV patients with RD dwelled longer in a state with more efficient functional integration and flexible interaction. These findings are helpful for us to better understand the underlying neural mechanisms of RD and potentially contribute to intervention development for BPPV patients with RD.

## Data availability statement

The original contributions presented in the study are included in the article/supplementary material, further inquiries can be directed to the corresponding authors.

## Ethics statement

The studies involving humans were approved by the Ethics Committee of the Second Affiliated Hospital of Xuzhou Medical University. The studies were conducted in accordance with the local legislation and institutional requirements. The participants provided their written informed consent to participate in this study.

## Author contributions

ZC: Data curation, Funding acquisition, Investigation, Methodology, Writing – original draft, Writing – review & editing. YC: Formal analysis, Methodology, Writing – original draft, Writing – review & editing. LX: Data curation, Investigation, Methodology, Project administration, Resources, Supervision, Writing – review & editing. X-EW: Conceptualization, Methodology, Project administration, Supervision, Validation, Writing – review & editing. YL: Data curation, Formal analysis, Investigation, Methodology, Writing – review & editing. CL: Data curation, Investigation, Methodology, Visualization, Writing – review & editing. DL: Data curation, Formal analysis, Investigation, Methodology, Writing – review & editing. HL: Formal analysis, Funding acquisition, Methodology, Project administration, Supervision, Writing – review & editing. LR: Funding acquisition, Investigation, Methodology, Project administration, Resources, Supervision, Writing – review & editing.

## Footnotes

^1^ http://www.nitrc.org/projects/gretna

^2^ http://www.fl.ion.ucl.ac.uk/spm/software/spm12

^3^ http://mialab.mrn.org/software/gift

^4^ http://research.ics.tkk.f/ica/icasso
